# The human factor

**DOI:** 10.2471/BLT.15.030215

**Published:** 2015-02-01

## Abstract

Cheikh Niang tells Fiona Fleck why listening to people and helping them adapt their customs are essential in the fight against Ebola in western Africa.

Q: You were invited to study the communities affected by Ebola in Sierra Leone and Mali last year and have since published a number of reports on these. What insights can you provide as a medical anthropologist?

A: Since Ebola virus disease was first identified in the Democratic Republic of the Congo in 1976, epidemiological analysis has shown that cultural practices with regard to physical contact with people who are sick are key factors in its spread. To tackle the disease, we not only need to know how to manage it clinically, but also how people perceive it – in order to develop successful health interventions. By looking at what communities are doing with the sick, by analysing relationship ties and interaction between people sick with Ebola and their families, we can find out why certain people are at more risk of infection. In the current epidemic, it was clear that the biomedical approach alone could not resolve many thorny issues and that a socio-anthropological approach was also needed.

Q: As one of the few medical anthropologists on Ebola outbreak in western Africa, what did you find during your field trip to Kailahun and Kenema districts of Sierra Leone?

A: People were afraid, and this fear, combined with a loss in confidence in the health system, was hampering efforts to stop the outbreak. In some cases fear led to denial that Ebola existed, or to a stigmatization of those who were infected and of their families. Sometimes fear led to violence against those trying to explain to the community how to avoid infection, and to attacks on Ebola treatment units. There was fear on all levels: in the community, in the health systems and in the political leadership. So we had to break this down and analyse it to find ways to overcome it. In the early days of the epidemic some people thought that Ebola had been brought into the country by political forces as a tool for domination or to make money. Things have improved since then, now that people have a better understanding of the disease, how to prevent infection and its origins.

“In some cases fear led to denial that Ebola existed.”

Q: Why the loss in confidence?

A: People perceived Ebola treatment units as meaning certain death, not giving hope – as they should do. The health system was seen as propagating the disease. To overcome this, the health system must acknowledge the limits of its knowledge and responses from the outset. When I visited villages in the two districts in Sierra Leone, I found that that there was considerable resistance to attempts to bring the outbreak under control. Resistance is a belief and it is always hidden. Medical experts don’t have the tools or the expertise to deal with it. They only see things on the surface and failed to see the underlying cultural realities. I found buckets of chlorine for hand sanitization in the places, doorways etc., that were controlled by men, not women. Women had not been consulted on their position but given their caring role for the sick, they were particularly vulnerable to infection. I also found that people were fed up with being told about hand sanitization, they knew how Ebola is transmitted but wanted to express themselves, be heard and take charge of their health matters and not be told how to do this in a paternalistic way.

Q: How can you restore that loss of confidence?

A: The anthropological approach helped rebuild confidence in the health services by providing training for health workers and others from the health system in listening to people and letting them express their concerns. We also helped health workers overcome their own fear of Ebola, which also contributed to the lack of public confidence in the health system. By overcoming their fears and having the courage to work with Ebola patients, health workers have emerged as the heroes in this epidemic. So confidence has been restored through a combination of listening to people and a determination to join in the community's struggle for their health. 

Q: We hear that communities affected by the Ebola epidemic in western Africa have changed many of their customs with regard to the sick and the deceased by avoiding physical contact. How did you and your colleagues help them to achieve that change?

A: The anthropological approach helped these communities preserve important cultural concepts while changing practices that were risky in the context of the epidemic. For example, the important concept of empathy with the sick person, expressed by touching them, can be transformed into encouraging families not to touch the sick people to protect the household and the community and to take the sick to treatment centres. Mourners in Sierra Leone traditionally wash, rub and touch the body of the deceased. This led to many infections earlier in the epidemic, when there was less awareness of how the virus is transmitted. The anthropological approach has helped communities reconnect with their traditional concept of death, while adapting the rituals surrounding death. The frustration at being unable to touch the deceased body at their funeral is alleviated by a new funeral rite, in which mourners ask the deceased for forgiveness. In Mali, we identified funeral rites that apply in special circumstances. Mourners do not wash the deceased body or touch it as they would with a “normal death” and applying this to Ebola deaths helped them accept the idea that these deaths require different treatment. In Mali, we – anthropologists – met the highest Islamic authorities in the land who helped to encourage the affected communities to make the necessary changes in their funeral rites to prevent further infections. We also gained the support of religious leaders to disseminate messages to communities about the importance of prevention and contact tracing.

Q: You are one of a group of international experts advising WHO on how to ensure the success of the clinical trials of vaccines, therapeutics and diagnostics for Ebola virus disease in western Africa. What is your role?

A: I have an advisory role. With regard to clinical trials, I attended several WHO meetings where we discussed trial design. Scientists argued that randomized-controlled trials would generate the most reliable and robust data on the efficacy of these interventions. But, although this design is considered the gold standard for generating strong scientific evidence, its feasibility would be limited by the same fear and resistance that I have described. In some places during the epidemic, quarantine has been enforced by the military. When you have a confrontation between the health system and the communities they serve, people feel powerless to make their voices heard and, in that context, when you conduct a randomized-controlled trial, people may ask: “Why have some people been selected to receive treatment, while others have not?” From the community’s perspective you have a huge ethical issue: treatment is available, but some people are not getting it because of the researcher’s need for data. For the community, it seems that data have more value than human life. At those meetings, I raised my voice about this, saying that we urgently needed social scientists and anthropologists on the ground to conduct consultations with members of the community so that they can provide their input in plans for clinical trials.

“For the community, it seems that data have more value than human life.”

Q: What were the main findings from your trip to Mali, where the outbreak was small and quickly contained? How did these findings contrast with those in Sierra Leone?

A: In Mali, the epicentre of the outbreak was within families – not whole communities – and the goal was to contain it within those families. To prevent relatives infecting each other, we had to listen to those who were most affected, so that their voices could be heard, and give a more positive image to the measures – such as contact tracing and isolation – needed to control the outbreak. The anthropological approach helped to encourage relatives who had come into contact with the sick and deceased to cooperate with isolation measures. We worked with one family of 70 people. About 25 of them refused to cooperate with measures to control transmission of the disease, but by talking to them and listening to their concerns, that resistance came down to zero refusals. We did similar work with communities talking and listening to them to help remove the stigma attached to people with Ebola. This helped rebuild social cohesion in communities divided by Ebola. In Mali, we helped health workers overcome their own fears so that they could work more closely with the communities affected by Ebola. 

Q: How did you become interested in medical anthropology?

A: I discovered medical anthropology while studying at Michigan State University as part of my doctoral thesis on sexuality and the HIV epidemic. These courses helped me understand better the need to integrate the human, social, cultural and political dimensions of our responses to an epidemic. I gained a better understanding of how gender, social and political factors influence the course of an epidemic. Anthropology allows us to connect the medical responses with social and political dynamics in some of the most vulnerable communities.

Q: Over the years much of your research has been on highly stigmatized communities in Africa, and, in particular, how to be more effective in providing prevention and care for HIV and other sexually transmitted infections for men who have sex with men. How can health services overcome the barriers of prejudice and discrimination?

A: We found that by providing counselling services and support on HIV and other sexually transmitted infections for the most stigmatized communities, we also learned how to better respond to the epidemic in society as a whole. We need interdisciplinary approaches – combining the biomedical with socio-anthropological approaches – to deal with these complex health problems, where the human factor can be decisive. Like Ebola, HIV infection is a highly stigmatized disease and the same concept of providing listening spaces for the individuals and communities affected applies. Health services need to be more proactive in terms of sending staff with psycho-social skills to work with the Ebola-affected communities, otherwise these communities become stigmatized and excluded, and reject official responses to the epidemic.

